# A Method of Calibration for the Distortion of LiDAR Integrating IMU and Odometer

**DOI:** 10.3390/s22176716

**Published:** 2022-09-05

**Authors:** Qiuxuan Wu, Qinyuan Meng, Yangyang Tian, Zhongrong Zhou, Cenfeng Luo, Wandeng Mao, Pingliang Zeng, Botao Zhang, Yanbin Luo

**Affiliations:** 1 School of Automation, Hangzhou Dianzi University, Hangzhou 310018, China; 2 Electric Power Research Institute of State Grid Henan Electric Power Company, Zhengzhou 450018, China

**Keywords:** motion distortion, IMU, sensor fusion, odometer, ICP

## Abstract

To improve the motion distortion caused by LiDAR data at low and medium frame rates when moving, this paper proposes an improved algorithm for scanning matching of estimated velocity that combines an IMU and odometer. First, the information of the IMU and the odometer is fused, and the pose of the LiDAR is obtained using the linear interpolation method. The ICP method is used to scan and match the LiDAR data. The data fused by the IMU and the odometer provide the optimal initial value for the ICP. The estimated speed of the LiDAR is introduced as the termination condition of the ICP method iteration to realize the compensation of the LiDAR data. The experimental comparative analysis shows that the algorithm is better than the ICP algorithm and the VICP algorithm in matching accuracy.

## 1. Introduction

Exact pose estimation is the key technology for mapping, location, and navigation in the field of the mobile robot [1], which can provide the message of the robot’s position and gesture in real time. Sensors used to obtain robot pose estimates include LiDAR, cameras, wheel encoders, IMUs, etc. According to the different sensors the robot is equipped with, SLAM technology is divided into visual SLAM and laser SLAM. Although the sensor used in visual SLAM has low cost and rich image information, it has a great impact on the normal operation of the camera under weak- or no-light conditions. What is more, because the image information is too rich, the algorithm requires high processor performance. Now, the mainstream mobile robots are still dominated by laser sensors [2], such as the unmanned delivery vehicle of JD and the “prime” unmanned delivery vehicle of Amazon.

A 2D LiDAR estimates the pose of the sensor by scan-matching two adjacent frames of laser data [3]. However, only relying on 2D laser SLAM to estimate the pose of the robot has many limitations. The frequency of the system output estimated pose is low, and the running time becomes longer, which will generate a large cumulative error and eventually affects the positioning and map construction of the robot. A cartographer algorithm [4] is developed using a SICK radar, and the frame rate can reach more than 100 Hz. The motion distortion can be ignored, so there is no distortion correction algorithm module. However, the frame rate of most LiDAR is around 10 Hz. Without distortion correcting, there will be distortion error appearing in LiDAR data, which is hard to eliminate through loopback detection and back-end optimization, etc. The research on this issue has great practical significance. Many domestic and foreign works have been conducted on removing motion distortion and false match of LiDAR data in recent years. Yoon et al. [5] proposed an unsupervised parameter learning in the Gaussian variational inference setting, which combines classical trajectory estimation of mobile robots and deep learning on rich sensor data to learn a complete estimator via the deep network. However, it requires a large amount of calculation, the captured laser data cannot complete feature extraction or matching when the environment is not clearly structured, and the real-time and robustness are poor. Therefore, it is only suitable for small indoor scenes with clear structure instead of open large outdoor scenes. Hyeong et al. [6] proposed an ICP (Iterative Closest Points, iterative closest point) outlier rejection scheme to compare the laser data of the scanned environment and select matching points and reject the algorithm that does not match parts. The ICP algorithm needs to be provided with an initial value, and the matching accuracy of the ICP algorithm directly depends on whether the initial value is accurate. However, in the process of acquiring the surrounding environment, the laser is often accompanied by the motion of the robot. Especially when the laser frame rate is small, the captured laser data will produce motion distortion, and there will be a large error with the real environment over time. Xue et al. [7] proposed a simultaneous fusion of IMU, wheel encoder, and LiDAR to estimate the own motion of a moving vehicle. However, this method does not propose a countermeasure for discontinuous laser scanning. Bazet and Cherfaoui [8] proposed a method for correcting errors caused by time stamp errors during sensor data acquisition, but this scheme assumes that the scanning angle of the laser is fixed and the quadratic interpolation assumption is too simplistic, which cannot meet the complex outdoor environment. Hong et al. [9] proposed a new approach to enhancing ICP algorithms by updating speed, which estimates the speed of the LiDAR through ICP iterations, and uses the estimated speed to compensate for scan distortion due to motion. Although it considers the motion of the robot into consideration, its assumption of uniform motion is too ideal; for low-frame rate LiDAR, the assumption of uniform motion does not hold.

Aiming at the above problems, this paper proposes an improved algorithm for estimated speed scan matching that integrates an IMU and odometer. This algorithm is called Iao_ICP (ICP that integrates IMU and Odometer) in this paper. The main contributions of this paper are as follows: (1) The algorithm uses the linear interpolation method to obtain the pose of LiDAR, which solves the alignment problem of the discontinuous laser scan data. (2) The data fused by the IMU and the odometer provides a better initial value for the ICP, and the estimated speed of the LiDAR is introduced as the iterative value of the ICP method to realize the termination condition of LiDAR data compensation.

The rest of the paper is organized as follows: Firstly, the causes of motion distortion in the traditional ICP algorithm are analyzed. Secondly, the incremental information of the wheel odometer and the angular velocity information of the IMU are integrated into the pose estimation. Finally, through data sets and physical experiments, the effectiveness of the proposed algorithm in removing motion distortion and improving the accuracy of map construction is demonstrated.

## 2. Causes of LiDAR Motion Distortion

The mechanical LiDAR is driven by an internal motor to rotate the radar ranging core 360° clockwise to obtain the surrounding environment data. Each frame of laser data is encapsulated by the data information obtained by a certain number of discrete laser beams, and the laser data of each frame is not obtained instantaneously. The data distortion of LiDAR is related to the motion state of the robot which carries LiDAR. When laser scanning is accompanied by the motion of the robot, the laser data of each angle is not obtained instantaneously. When the scanning frequency of the LiDAR is relatively low, the motion distortion of the laser frame caused by the motion of the robot cannot be ignored [10].

**Figure 1:**
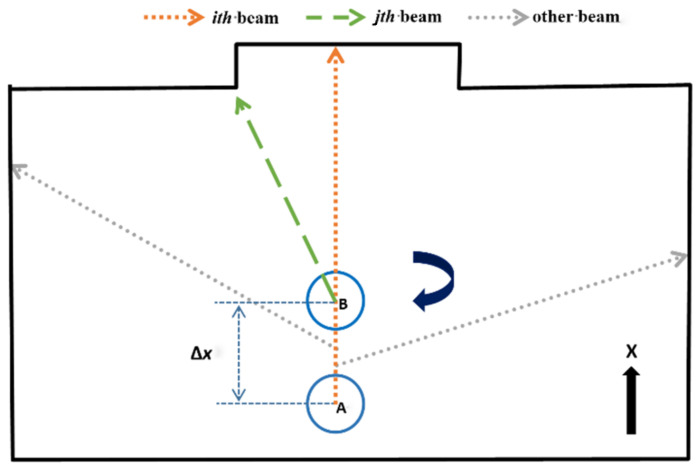
The acquisition process of one frame of LiDAR data.

The current domestic LiDAR rotation frequency is about 5–10 Hz. When the robot carrying the LiDAR is stationary, the measurement data of the LiDAR has no error, but in the SLAM system, the robot is often in a state of motion. Take the environment shown in Figure 1 as an example. It can be seen that the distance data of each laser beam are collected in different poses, as shown in the pose of points A and B. Suppose the robot is moving at a constant speed, the solid curved arrow indicates that the LiDAR rotation direction is clockwise, and the solid long straight arrow indicates that the LiDAR moves from point A to point B along the X direction. Then, in the case of no motion distortion correction during this period, the LiDAR data will have a motion distortion error of Δx.

**Figure 2:**
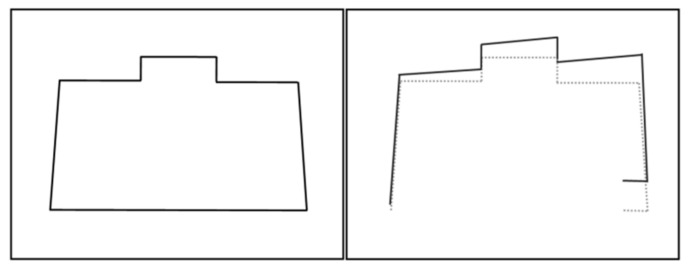
LiDAR motion distortion.

As described above, when the robot obtained a frame of LiDAR data, the laser is obtained at point A, and the laser is obtained at point B. However, when general LiDAR drives package data, it is assumed that all laser beams of a frame of LiDAR data are obtained in the same pose and instantaneously, that is, all laser beams are obtained from point A data. Its pose actually produces motion changes, and each laser point is generated on a different reference pose, which eventually causes the environmental distortion of the laser collection. As Figure 2 shows, the left picture is the actual environment, while the dotted line in the picture on the right is the true value, and the solid line is the LiDAR data with motion distortion.

## 3. Principle of ICP Algorithm

The ICP algorithm [11] was first developed by Beals and McKay in 1992. The ICP algorithm is essentially an optimal registration method based on the least-squares method. ICP first matches each point of the target laser data with the closest point of the reference laser data and finds the rotation matrix R and translation matrix p, which are used to convert the two. Afterward, the laser matching is iteratively optimized by repeatedly generating pairwise closest points until the convergence accuracy requirements for correct registration are met. The ICP algorithm first needs to determine an initial pose, and the selected initial value will have an important impact on the final registration result. The algorithm may fall into a local optimum instead of a global minimum if the initial value is not chosen properly.

Given $X=\left\{x_{1},x_{2},\cdots ,x_{N_{x}}\right\}$ as a frame of laser data, $P=\left\{p_{1},p_{2},\cdots ,p_{N_{p}}\right\}$ as the laser data of adjacent frames, and $T=\left\{T_{1},T_{2},\cdots ,T_{i}\right\}$ as the transformation matrix of laser data of adjacent frames, $x_{i}$ and $p_{i}$ indicate the coordinates of the laser spot, $N_{x}$ and $N_{p}$ indicate the number of laser dots, and i indicates the frame number of laser data. This paper defined a minimizing objective Function (1) to transform P through the coordinates, and cover the maximum to X [11].

(1) $$E\left(R,p\right)=\frac{1}{N_{p}}\sum_{i=1}^{Np}∥x_{i}-Rp_{i}-t{∥}^{2}$$

The resulting transformation matrix *T* can be described as (2):

(2) $$T=\left[\begin{array}{cc} R & p\\ 0 & 1 \end{array}\right]$$

The processing steps of the given objective function are shown as follows:

Step1: Solving the mean value of LiDAR data *X* and *P*:

$$U_{x}=\frac{1}{N_{x}}\sum_{i=1}^{N_{x}}X_{i},U_{p}=\frac{1}{N_{p}}\sum_{i=1}^{N_{p}}P_{i};$$

Step2: Remove the translation of LiDAR data *X* and *P* to distributed laser data around the mean value:

$$x_{i}^{\prime }=x_{i}-U_{x},p_{i}^{\prime }=p_{i}-U_{p}$$

Step3: Define matrix, and make SVD decomposition of it, where *H* is the matrix to be decomposed by SVD, *U* and *V* are the two non-singular matrices decomposed, and $\sigma_{1}$, $\sigma_{2}$, and $\sigma_{3}$ are the three singular values decomposed, respectively:

$$H=\overset{Np}{\underset{i=1}{\sum }}x_{i}^{\prime }p_{i}^{\prime T}=U\left[\begin{array}{ccc} \sigma_{1} & 0 & 0\\ 0 & \sigma_{2} & 0\\ 0 & 0 & \sigma_{3} \end{array}\right]V^{T}$$

Step4: Calculate the solution of the objective function:

$$R=UV^{T},\;p=u_{x}-Ru_{p}$$

**Figure 3:**
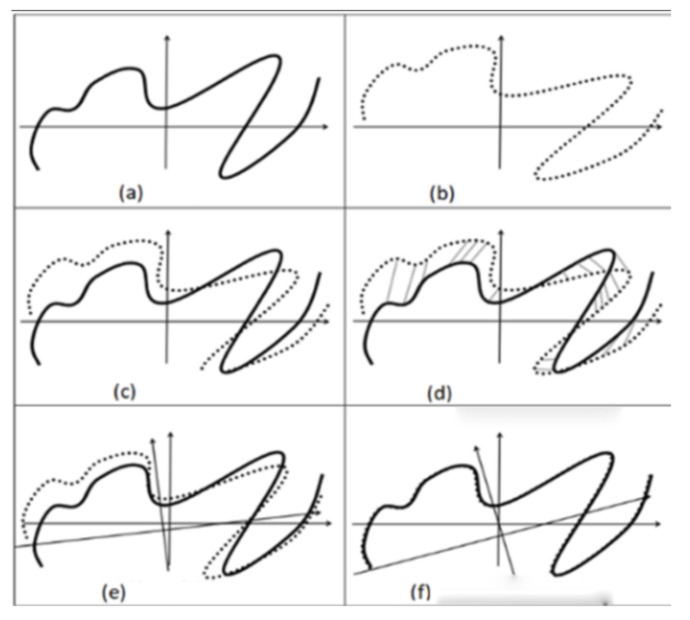
The principle of ICP algorithm. (**a**) Frame *i* (**b**) Frame *i* + 1 (**c**) Start matching (**d**) Find adjacent (**e**) First match (**f**) After multiple iterations of matching.

Since the ICP algorithm uses the closest point as the corresponding point, the initial result may be different from the real environment. However, the results converge to the base environment by repeating this process. The LiDAR scan data for frame *i*, namely, *X*, are shown in Figure 3a. The LiDAR scan data for frame *i* + 1, namely, *P*, are shown in Figure 3b. The first step of ICP iteration is shown in Figure 3c. The closest point between *X* and *P* is found as Figure 3d shows. The first matching estimated transformation and updated *P* by $p_{i}^{\prime }=T_{1}p_{i}$, which is shown in Figure 3e. The *X* and *P* matched after many iterations, as Figure 3f shows. Final pose estimation is solved through the transformation of $T=T_{n}T_{n-1}\cdots T_{2}T_{1}\left(i=1,\cdots ,n\right)$, namely:

(3) $$x_{i}=T_{n}T_{n-1}\cdots T_{2}T_{1}p_{i}=Tp_{i}$$

## 4. Estimation Speed Scan Matching Algorithm Based on IMU and Odometer

**Figure 4:**
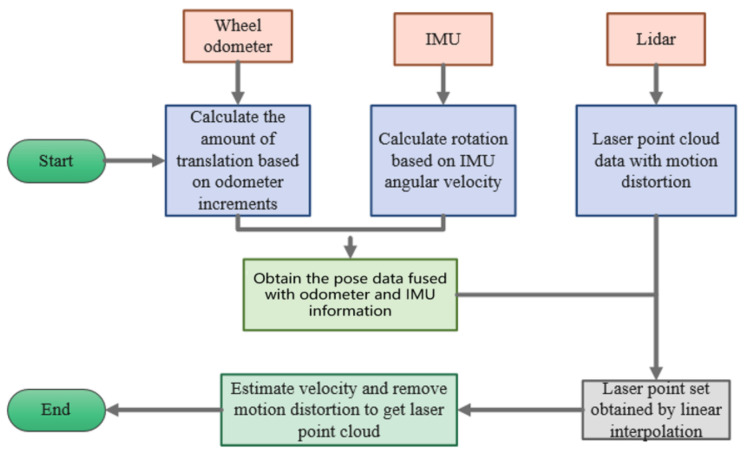
The architecture diagram of the Iao_ICP algorithm.

A wheeled odometer and IMU are introduced to compensate for motion distortion of laser data caused by robot moves. Direct measurement of displacement and angle information through a wheel odometer or direct measurement of angular velocity and linear acceleration through an IMU [12], then integrate them, respectively, to obtain the displacement and angle information. In the ideal conditions, the wheel odometer or IMU has a high-precision local pose estimation ability because of the high pose update frequency (higher than 200 Hz) of the above sensors, which can accurately reflect the motion of the robot in real time [13]. What is more, these two types of sensors are completely decoupled from the robot state estimation, which can prevent the introduction of errors. However, on the one hand, during the actual movement of the robot, the wheels will slip and the accumulated error will occur, which leads to a certain deviation in the obtained odometer angle data when only the encoder is used, and the error increases with the running time and the stroke increases. On the other hand, the linear acceleration accuracy of the IMU is poor, though it has high angular velocity measurement accuracy, and the local accuracy of the quadratic integral is still very poor, which leads to a certain deviation of obtained displacement data. Therefore, this paper proposes the Iao_ICP algorithm, and the algorithm framework is shown in Figure 4. First, the information of the IMU and the odometer is fused, and the pose of the LiDAR is obtained using the linear interpolation method to remove most of the motion distortion. Then, scan matching of LiDAR data is conducted using the ICP method. Data fused by the IMU and odometer provide a better initial value for ICP, and estimated speed is introduced as a termination condition for iteration of the ICP method [14]. The matching result is used as the correct value, and the error value of the odometer is obtained. The error value is evenly distributed to each point, and the position of the laser point is corrected again, so as to further determine the pose of the laser point.

### 4.1. Pose Estimation with Fusion of IMU and Odometer

The chassis control system of the mobile robot reads the IMU data and the odometer data. Each time the IMU data are read, the odometer data can also be obtained without considering the problem of time synchronization. That means the IMU pose queue and odometer pose queue maintain strict alignment, which can directly fuse both to generate a new pose queue. However, the update frequency of low-cost LiDAR is generally only 5–10 Hz, which leads to the new pose queue after fusion cannot maintain strict alignment with the pose queue of laser frames. Although there is no way to obtain the pose of the laser frame directly from the fused pose queue since the pose queues of the two are not strictly aligned, the pose of the laser frame can be obtained by linearly interpolating the fused pose queue. Below are the detailed steps to obtain the estimated pose based on the linear interpolation method by fusing the IMU and odometer data:

Step1: As the start time of the current laser frame, the end time of the current laser frame, and the time interval between two laser beams have been known. Odometer data and IMU data are stored in a queue in the same chronological order, and the team leader is the earliest. There are oldest odometer and IMU data timestamps, and latest odometer and IMU data timestamps. First, solve the new queue generated by fusing odometry and IMU data within the above timestamps. The fusion expressions are shown below:

(4) $$\left\{\begin{array}{c} Odom\_Imu\_List\left[i\right].x=OdomList\left[i\right].x\\ Odom\_Imu\_List\left[i\right].y=OdomList\left[i\right].y\\ Odom\_Imu\_List\left[i\right].\theta =ImuList\left[i\right].\theta \end{array}\right.$$

In the formula, $Odom\_Imu\_List\;\left[i\right]$ is the fused pose data at the $t_{i}$ moment, $OdomList\;\left[i\right]$ is the odometer pose data at the $t_{i}$ moment, $ImuList\;\left[i\right]$ is the IMU pose data at the $t_{i}$ moment, and x, y, and θ are the X-axis data, Y-axis data, and angle data in the pose data, respectively.

**Figure 5:**
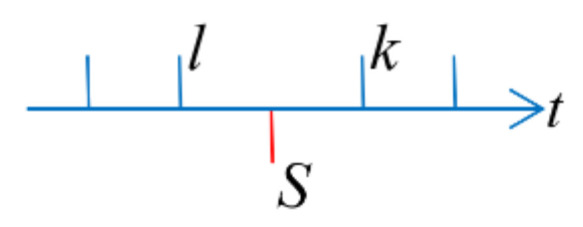
Linear interpolation of laser pose.

Step2: Solve the emission pose corresponding to each laser in the current frame of laser data, namely, to solve the robotic pose at the time of $\left\{t_{s},t_{s}+\Delta t,\cdots t_{s}+i\Delta t\cdots t_{e}\right\}$. It is reasonable to assume that the robot moves at a uniform speed during the data update of the fusion of two adjacent frames due to the high update frequency of odometer data and IMU data. Linear interpolation can be used on this assumption, as shown in Figure 5.

Suppose there are corresponding fused pose queues at the time of *l*, *k* for laser data, but not at the time of s, and the value of s is greater than l, and less than *k*. Then, solve the pose of robot $p_{s}$, $p_{m}$, $p_{e}$ corresponding to the three moments *t_s_, t_m_, t_e_* $\;(t_{s}<t_{m}<t_{e})$. The pose of the first laser beam can be calculated with the Formula (5). In the same way, the emission pose of the last laser beam and the laser beam at the middle time can be obtained.

(5) $$\left\{\begin{array}{c} p_{l}=Odom\_Imu\_List\left[i\right]\\ p_{k}=Odom\_Imu\_List\left[k\right]\\ p_{s}=p_{l}+\frac{p_{k}-p_{l}}{k-l}\left(s-l\right) \end{array}\right.$$

**Figure 6:**
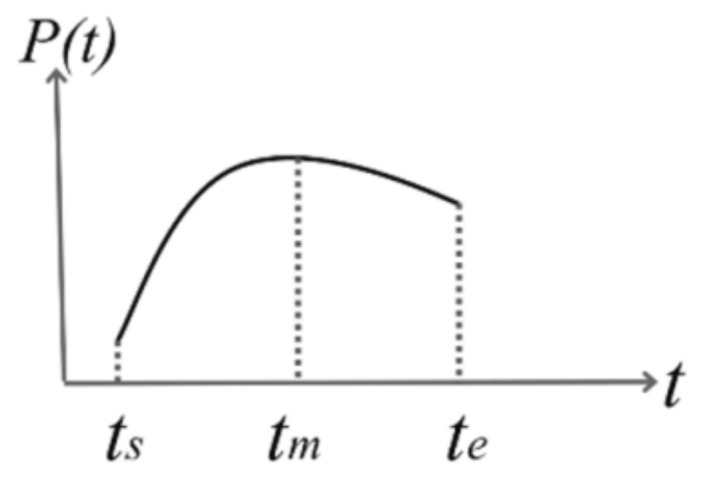
Pose function graph.

Step3: Following the method in the Step2, $p_{m}$ and $p_{e}$ can be solved. Further assumed, the robot performs uniform acceleration motion during a frame of laser data. Thus, the pose of the robot is a quadratic function of time, as Figure 6 shows. Thus, using the known robot pose $p_{s}$, $p_{m}$, $p_{e}$ as the independent variable, a quadratic curve function $P\left(t\right)=At^{2}+Bt+C\left(t_{s}<t<t_{e}\right)$ can be obtained by interpolation, and A, B, C are the coefficients of the quadratic function. Next, the value of every time $\left\{t_{s},t_{s}+\Delta t,\cdots t_{s}+i\Delta t\cdots t_{e}\right\}$ can be substituted into a curve, and the pose of each laser point data in global coordinate system $\left\{p_{t_{s},}p_{t_{s}+\Delta t},\cdots p_{t_{s}+i\Delta t}\cdots p_{t_{e}}\right\}$ can be obtained.

Step4: The relative pose (array form) of the laser point in the global coordinate system is converted into a pose change matrix. Then, convert the coordinate information in the radar coordinate system xi to the coordinates in the global coordinate system, as Formula (6) shows.

(6) $$x_{i}^{\prime }=V2T\left(p_{i}\right)x_{i}$$

In the above Formula (6), function $V2T\;\left(p_{i}\right)$ is a whole, indicating that the relative pose in the form of an array $p_{i}$ is converted into a pose transformation matrix in the form of a matrix. By the coordinate information in radar system $x_{i}$ left multiplication corresponding matrix $p_{i}$, the coordinate of the radar coordinate system $x_{i}$ can be translated into the coordinate in the global coordinate system $x_{i}^{\prime }$, because $p_{i}$ is the pose in the global coordinate system.

Step5: According to the coordinates of the scanning point corresponding to each laser beam in the global coordinate system $x_{i}^{\prime }$, the laser data of the laser scan point in the LiDAR coordinate system can be solved with Formula (7).

(7) $$\left\{\begin{array}{c} x_{i}^{\prime }=\left(p_{x},p_{y}\right)\\ range=\sqrt{p_{x}\cdot p_{x}+p_{y}\cdot p_{y}}\\ angle=\mathrm{atan}2\left(p_{y},p_{x}\right) \end{array}\right.$$

For the first equation above, $p_{x}$, $p_{y}$ are the coordinates of the ith frame of laser data in the LiDAR coordinate system on the X- and Y-axis, respectively.

For the second equation above, the coordinates $p_{x}$ and $p_{y}$ on the x and y axes of the laser $x_{i}$ frame in the laser coordinate system are known. The distance point $x_{i}$ from the origin of the laser coordinate system can be found according to the “Pythagorean Theorem”.

For the third equation above, $p_{x}$ and $p_{y}$ have been found, and the angle between point $x_{i}$ and X-axis can be solved according to inverse trigonometric functions. The specific implementation process of the algorithm is shown in Algorithm 1.
**Algorithm 1: A Pose Estimation Algorithm Based on IMU and Odometer**Input: Odometer pose queue $OdomList\left[i\right]$, IMU pose queue, and laser pose queue $x_{i}$Output: laser pose queue $X_{n}$1: ***for***
*i = 1:n **do*** 2: $Odom\_Imu\_List\left[i\right].x=OdomList\left[i\right].x$; $Odom\_Imu\_List\left[i\right].y=OdomList\left[i\right].y$; $Odom\_Imu\_List\left[i\right].\theta =ImuList\left[i\right].\theta$; //fuse the data of odometer and IMU pose queue, then put into $Odom\_Imu\_List\left[i\right]$ 3: **end for** 4: $p_{s}=LinerInterp\left(Odom\_Imu\_List\left[t_{s}\right]\right)$; $p_{m}=LinerInterp\left(Odom\_Imu\_List\left[t_{m}\right]\right)$; $p_{e}=LinerInterp\left(Odom\_Imu\_List\left[t_{e}\right]\right)$; //Perform linear interpolation on the fusion pose of the start, end and intermediate moments, LinerInterp() is function used to make linear interpolation 5: $P\left(t\right)=P\left(t\right)=At^{2}+Bt+C$; //Substitute $p_{s}$, $p_{m}$, $p_{e}$ into above formula in order, and the coefficients of quadratic curve functions A, B, C can be solved. 6: ***for***
*i = 1:n **do*** 7: $p_{i}=Ai^{2}+Bi+C$; //solve the pose of each laser point in global coordinate system $p_{i}$ 8: ${x^{\prime }}_{i}$ = $V2T\left(p_{i}\right)x_{i}$ = $\left(p_{x},p_{y}\right)$; //obtain the pose of each laser point in the global coordinate system ${x^{\prime }}_{i}$ 9: $X_{n}=\left(range,angle\right)=\left(\sqrt{p_{x}*p_{x}+p_{y}*p_{y}},\mathrm{atan}2\left(p_{y},p_{x}\right)\right);$ //compose a new laser point set $X_{n}$ 10: **end for**

### 4.2. Estimated Velocity and Laser Data Pose Compensation

To remove the motion distortion of the laser point cloud data, the speed of the robot needs to be estimated. Since the scanning period of LiDAR is about 0.1 s, it can be assumed that the speed of the robot is constant during this scanning period, and $V_{i}$ is used to indicate the velocity in the LiDAR coordinate system at $t_{i}$ time. Firstly, estimate the velocity $V_{i}$ from the relative motion transformation between two adjacent frames of laser data $X_{i}$ and $X_{i-1}$, supposing that n indicates the number of laser points of laser data $X_{i}$. The time interval between two adjacent frames of laser points is Δt. $x_{0\;}$, $x_{1\;}$, ⋯, $x_{n\;}$ is the laser point of laser $X_{i}$, $t_{x_{j}}-t_{x_{j-1}}=\Delta t_{s}\;\left(j=0,1,\cdots ,n-1\right)$.

Therefore, the estimated velocity $V_{i}$ is:

(8) $$V_{i}=\frac{T2V\left(T_{i-1}^{-1}T_{i}\right)}{\Delta t}\approx \frac{1}{\Delta t}\mathrm{lg}\;T_{i-1}^{-1}T_{i}$$

In Formula (8), $T_{i-1}^{-1}T_{i}$ is a whole, indicating the pose difference of the robot from *i*−1 time to *i* time in the radar coordinate system, and $T2V\left(T_{i-1}^{-1}T_{i}\right)$ indicates a way to convert the pose difference from matrix form to array form.

The pose of frame *i* and laser point *j* is:

(9) $$T\left(t_{i}+j\Delta t_{s}\right)=T_{i}\cdot V2T\left(V_{i}\cdot j\Delta t_{s}\right)=T_{i}e^{j\Delta t_{s}}V_{i}$$

In Formula (9), $j\Delta t_{s}$ is the duration of laser point cloud data in frame *i* from laser point 0th to laser point *j*.

$V_{i}$ is the origin velocity of laser point data in frame *i*.

$V_{i}\cdot j\Delta t_{s}$ is the pose difference of frame *i* laser data cloud from laser point 0th to laser point *k*.

$V2T\left(V_{i}\cdot j\Delta t_{s}\right)$ is the conversion of relative pose difference from array form to matrix form.

$T_{i}\cdot V2T\left(V_{i}\cdot j\Delta t_{s}\right)$ is to obtain the pose of laser point *j* in frame *i* by using frame *i* of laser point cloud data right-multiplied by the pose difference from the initial pose of the 0th laser point.

Substitute the above Formula (9) into Formula (3), the laser point cloud data collection $X_{i}$ is converted into ${\bar{X}}^{*}$, and ${\bar{X}}^{*}$ is the laser point cloud data collection after speed compensation.

(10) $${\bar{X}}^{*}=\left\{e^{j\Delta t_{s}V_{i}}p_{j}|j=0,1,\cdots ,n\right\}$$

For some types of LiDAR, it takes 100 ms to perform a scan with a scan angle of 360°, which takes the estimation of robot motion later than the actual movement. To prevent this kind of delay, a backward compensation scheme can be used. Take the time corresponding to the last laser point as the reference time, the corresponding time of each laser point can be converted. With the above conditions, Formula (9) can be revised into:

(11) $$T\left[t_{i}-\left(n-j\right)\Delta t_{s}\right]=T_{i}e^{\left(n-j\right)\Delta t_{s}\left(-V_{i}\right)}$$

Formula (10) can be revised into:

(12) $$\bar{X}=\left\{e^{\left(n-j\right)\Delta_{s}\left(-V_{i}\right)}x_{j}|j=0,1,\cdots ,n\right\}$$

The specific implementation process of the algorithm is shown in Algorithm 2.
**Algorithm 2: Estimating velocity and removing motion distortion from laser point cloud data combined with ICP**Input: the queue of laser pose $X_{n}$
Output: motion transformation matrix of adjacent laser frames T1: $V=V_{i}$ //speed initialization 2: do 3: $T_{\Delta \mathrm{ts}}=e^{\Delta \mathrm{ts}\left(-V_{i}\right)}$ //the motion transformation matrix T is estimated by the speed of the two adjacent frames of laser light 4: for j=1 :n do //traverse all laser points in the current laser frame 5: $T_{j\Delta \mathrm{ts}}=T_{\left(j-1\right)\Delta \mathrm{ts}}T_{\Delta \mathrm{ts}}$ //calculate the motion transformation matrix of each laser point 6: ${\bar{x}}_{i_{j}}=T_{j\Delta \mathrm{ts}}x_{i_{j}}$ //Motion transformation for each laser point 7: end **for**
8: $T=\mathrm{ICP}\left({\bar{X}}^{-1},{\bar{X}}_{i},T\right)$ //iterative matching via ICP 9: $V=V_{i}$ //renew the value of velocity 10: $V_{i}=1/\Delta \mathrm{lg}\;T$ //do the next round of speed estimation 11: $While\;\left|\left|V-V_{i}\right|\right|>e$ //when the speed error value is greater than the threshold e, execute the loop

## 5. Positioning Accuracy Evaluation of Laser Odometry after Motion Distortion Calibration

**Figure 7:**
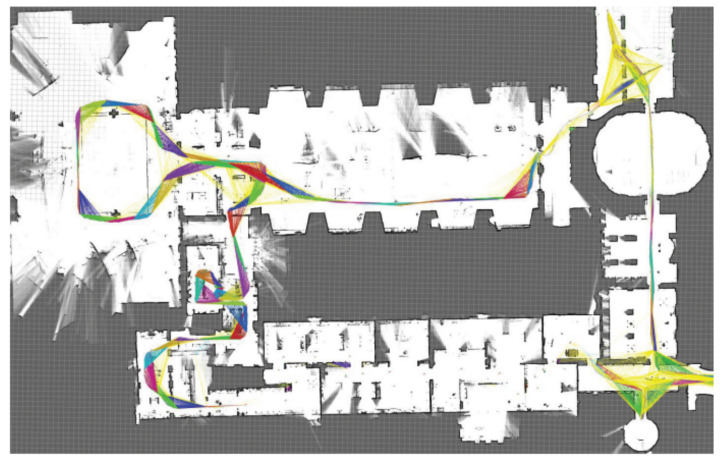
Mapping based on the b0_2014_07_11_11_00_49 sequence.

This experiment utilizes the sequences b0_2014_07_11_10_58_16 (denoted as ①), b0_2014_07_11_11_00_49 (denoted as ②), and b0_2014_07_21_12_42_53 (denoted as ③) in the Cartographer public dataset. The laser odometry accuracy of the Iao_ICP algorithm and the original Cartographer algorithm is quantitatively evaluated by executing this. Figure 7 shows the mapping effect of the Iao_ICP algorithm on the sequence. The processor of the test equipment is Intel (R) Core (TM) i5−5200 CPU 2.20 GHz and it has 8 GB RAM.

The analysis is performed by comparing the data calculated by the Iao_ICP algorithm with the Cartographer data set. Table 1 lists the absolute trajectory errors calculated by these two algorithms [15]. In addition, the Iao_ICP algorithm was used to calculate the relative trajectory error and compared with the relative trajectory error of the original Cartographer algorithm.

**Figure 8:**
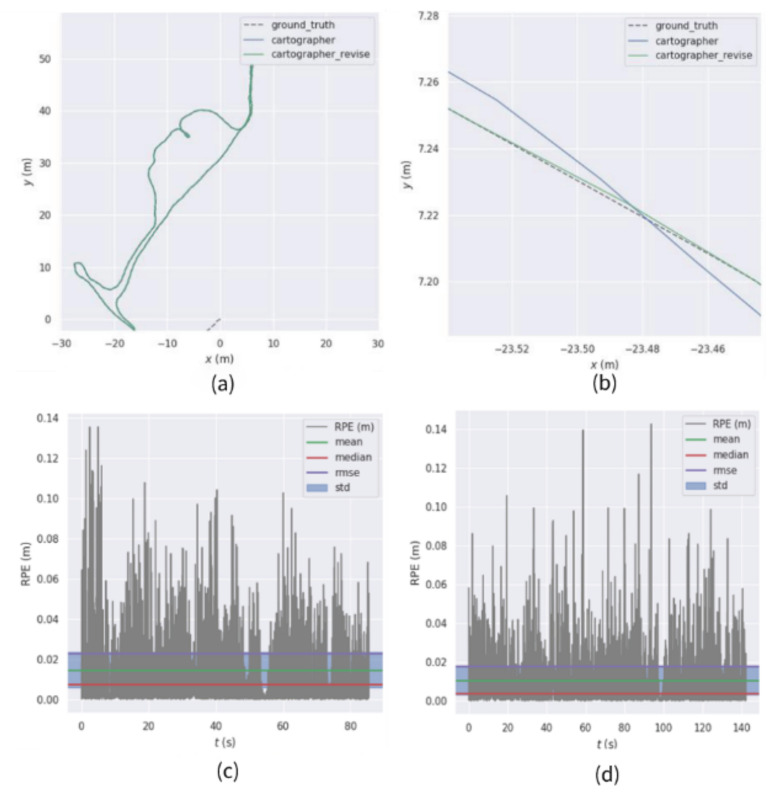
Comparison of relative trajectory errors of sequence ①. (**a**) Cartographer, improvement scheme, and real trajectory comparison (**b**) Local trajectory map (**c**) Absolute trajectory error of Cartographer (**d**) Absolute trajectory error of the improved scheme.

Using sequence ① for testing, the comparison of the relative trajectory error results obtained is shown in Figure 8.

**Figure 9:**
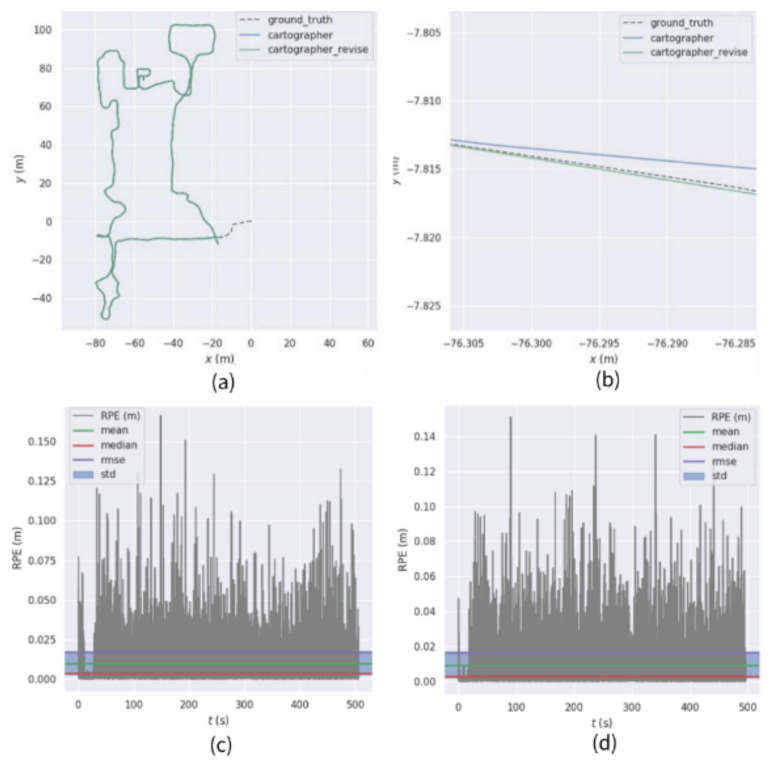
Comparison of relative trajectory errors of sequence ②. (**a**) Cartographer, improvement scheme, and real trajectory comparison (**b**) Local trajectory map (**c**) Absolute trajectory error of Cartographer (**d**) Absolute trajectory error of the improved scheme.

The obtained comparison of relative trajectory error results is shown in Figure 9 by using a sequence for testing.

**Figure 10:**
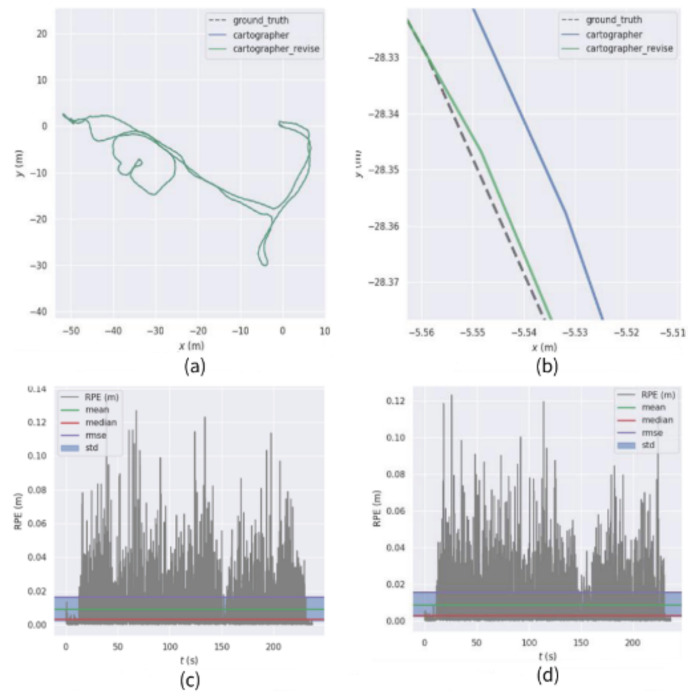
Comparison of relative trajectory errors of sequence ③. (**a**) Cartographer, improvement scheme, and real trajectory comparison (**b**) Local trajectory map (**c**) Absolute trajectory error of Cartographer (**d**) Absolute trajectory error of the improved scheme.

The obtained comparison of relative trajectory error results is shown in Figure 10 by using a sequence for testing.

From the sequence ① test results, it can be seen that, from the RMSE index, the root-mean-square error of the Iao_ICP algorithm is 0.0179 m, and the root-mean-square error of the original Cartographer algorithm is 0.0230 m. Compared with the original Cartographer algorithm, the root-mean-square error of the Iao_ICP algorithm is reduced by 22.06%. The average error of the Iao_ICP algorithm is 0.0044 m smaller than that of the original Cartographer algorithm. The maximum absolute trajectory error of the original Cartographer algorithm is 0.1428 m in this sequence, and the maximum absolute trajectory error of the Iao_ICP algorithm is 0.1357 m. The minimum absolute trajectory error of the Cartographer original algorithm is 0.0024 m, and the minimum absolute trajectory error of the Iao_ICP algorithm is 0.0011 m. It can be seen from the above data that the Iao_ICP algorithm has a smaller relative trajectory error than the original Cartographer algorithm in sequence ①.

## 6. Physical Experiment Analysis

This experiment uses a small wheeled differential car as the mobile robot platform.

**Figure 11:**
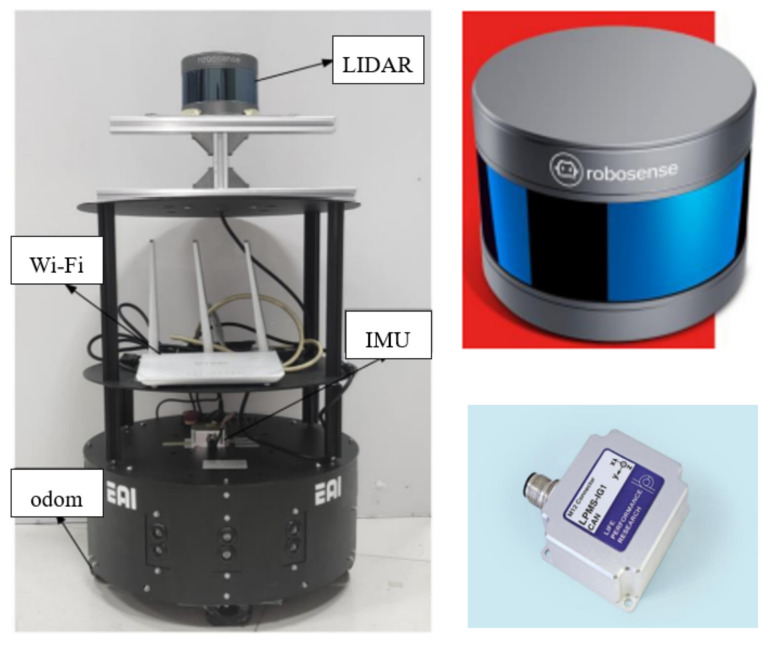
Mobile experiment platform.

As shown in Figure 11, the platform configuration is as follows: wheeled robot, embedded development board, 16-line RS-LIDAR-16 scanner, IPMS-IG IMU. Among them, the wheeled robot is driven by four wheels and two motors. The embedded development board uses STM32f103 as the main controller, and it is also equipped with a motor driver module and an MPU6050 module. RS-LiDAR-16 adopts a hybrid solid-state LiDAR, which integrates 16 laser transceiver components. The measurement distance is up to 150 m, the measurement accuracy is within ±2 cm, the number of output points is up to 300,000 points/s, the horizontal angle is 360°, the vertical measurement is 360°, and the angle is ±15°. IMU integrates three-axis acceleration and angular velocity sensors, which can measure the real-time pose of the robot, and has the advantages of high precision, high frequency, low power consumption, and strong real-time performance. This experiment realizes the conversion of 3D LiDAR to 2D LiDAR by projecting the 16-line data of 3D LiDAR onto a fixed plane. Since the real motion trajectory of the robot cannot be accurately obtained in the real scene, this experiment judges and tests the cumulative error of the robot pose during the mapping process of the Iao_ICP algorithm according to the loopback effect. The movement of the robot is controlled by the handle in this experiment.

**Figure 12:**
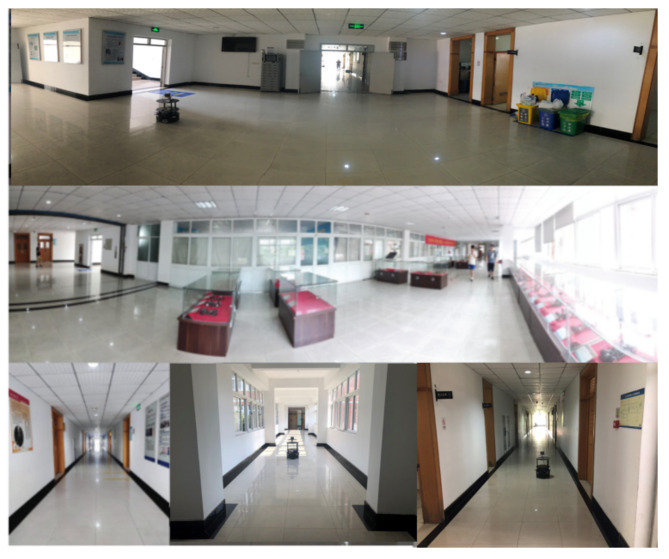
Experimental real scene.

The real environment is a rectangular hall corridor with a length of about 43 m, a width of about 51 m, and a building area of about 2193 m^2^, as shown in Figure 12 above. It is easy to measure the actual size of the object and compare the data with the mapping accuracy of the test algorithm. Due to the cabinets, building supports, stair entrances, elevator entrances, and other objects in the environment have a strong structure, the effectiveness and robustness of the algorithm for eliminating laser motion distortion and mapping accuracy can be tested in the above environment. There are also the following reasons: the test scene is relatively large, and there are long straight corridors, transparent glass, flowing crowds, and other factors in the environment that may easily interfere with the test of mapping. The smooth marble floor increases the accumulation of pose errors during the movement of the robot.

**Figure 13:**
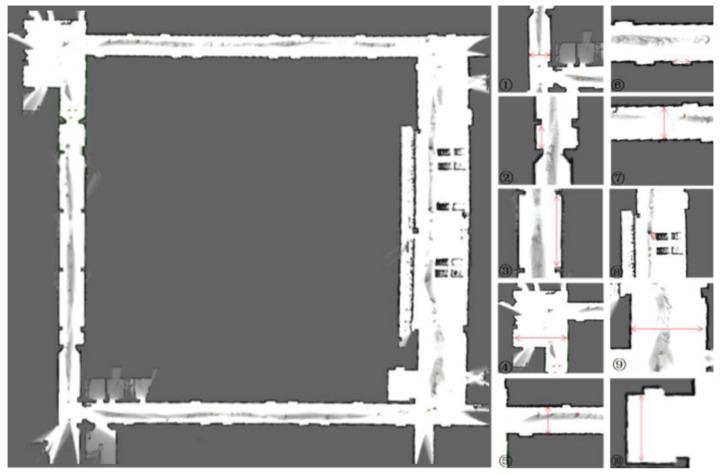
Mapping effect of Cartographer.

**Figure 14:**
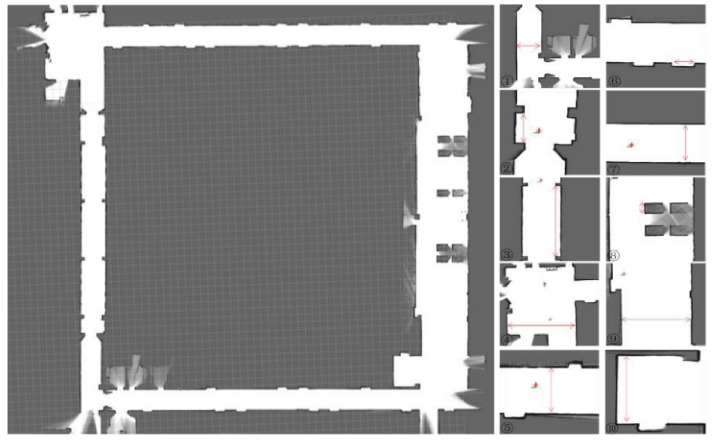
Mapping effect of Iao_ICP.

**Figure 15:**
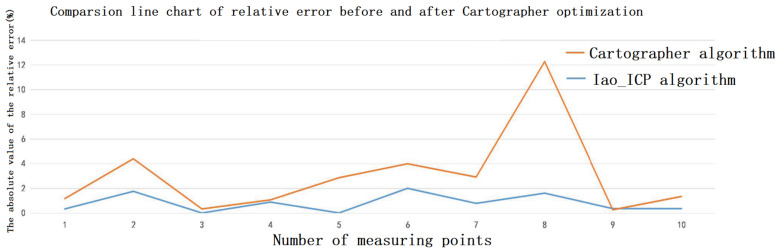
Line chart of relative error comparison of two algorithms.

To compare the mapping accuracy of the Iao_ICP algorithm and the original Cartographer algorithm, 10 highly structured objects were selected in the test scene for measurement and analysis. Figure 13 and Figure 14 are the mapping effect of the original Cartographer algorithm and the mapping effect of the Iao_ICP algorithm. First, the actual size of the object is measured by a handheld laser rangefinder. The map measurements displayed in the rviz plugin for algorithmic mapping are measured. Finally, the relative error and absolute error of the two algorithms are calculated. The measurement data and error values of the above two algorithms are shown in Table 2 and Table 3 below. Figure 15 is a comparison chart of the relative error of the two algorithms.

It can be seen from Figure 13 and Figure 14 that the original Cartographer algorithm has a large pose error product in this experimental scene. Although a loop can be formed, the effect of eliminating local errors on the map is not good. The Iao_ICP algorithm removes motion distortion from most laser data by fusing wheel odometer and IMU information. At the same time, the laser scan data are compensated by estimating the speed of the robot and ICP algorithm. The Iao_ICP algorithm not only effectively removes motion distortion, but also eliminates the accumulation of pose errors caused by tire slippage during robot motion. Figure 14 shows that the map constructed by the Iao_ICP algorithm has no confusion, no burrs, and clear structural features. It can clearly express the surrounding environment information, and the map ghost is small. It can be seen that the mapping effect of the Iao_ICP algorithm is better than that of the original Cartographer algorithm. Combined with the error data analysis in Table 2 and Table 3, and Figure 15, it can be seen that the average relative error of the Iao_ICP algorithm is much smaller than that of the original Cartographer algorithm, and the relative error is mostly concentrated below 1%. The error is stable, and there is no mutation. 

## 7. Conclusions

For the problem of removing laser motion distortion, in the case of wheel slippage and accumulated error, the traditional method of directly measuring displacement and angle information based on the wheel odometer, and the odometer angle data obtained by the encoder, will have a certain deviation. In addition, with the traditional method of directly measuring the angular velocity and linear acceleration based on the inertial navigation unit, and then integrating the displacement and angle information, due to the poor accuracy of the linear acceleration of the IMU, the local accuracy of the quadratic integration is still very poor. Therefore, the displacement data obtained will also have a certain deviation. The Iao_ICP algorithm proposed in this paper uses the linear interpolation method to obtain the pose of the LiDAR, which solves the alignment problem of discontinuous laser scan data. Data fused by IMU and odometer provide a better initial value for ICP. The estimated speed is introduced as the termination condition of the ICP method iteration to realize the compensation of the LiDAR data. The experiment uses a small wheeled mobile robot to collect data and compare and analyze results in a corridor environment to verify the original Cartographer algorithm and the Iao_ICP algorithm. Finally, the experimental data show that the algorithm proposed in this paper can effectively remove laser motion distortion, improve the accuracy of mapping, and reduce the cumulative error.

## Figures and Tables

**Table 1:** Comparison results of absolute trajectory error between Iao_ICP algorithm and Cartographer algorithm.

Sequence	Algorithm	RMSE (m)	Average (m)	Maximum (m)	Minimum (m)
①	Iao_ICP	0.0179	0.0103	0.01356	0.0011
	Cartographer	0.023	0.0147	0.01428	0.0024
②	Iao_ICP	0.0166	0.0091	0.1510	0.0008
	Cartographer	0.0197	0.0096	0.1663	0.0011
③	Iao_ICP	0.0158	0.0089	0.1233	0.0003
	Cartographer	0.0193	0.0092	0.1349	0.0012

**Table 2:** Cartographer original algorithm mapping error table.

Measuring Point	Measured Value (cm)	Figure Measured Values (cm)	Absolute Error (cm)	Relative Error (%)
1	284.700	288.074	−3.374	−1.185107
2	195.000	186.400	8.600	4.410256
3	712.200	709.709	2.491	0.349761
4	812.000	803.200	8.800	1.083743
5	271.000	263.200	7.800	2.878228
6	136.300	130.840	5.460	4.005869
7	272.300	264.320	7.980	2.930591
8	76.500	85.895	−9.395	−12.281045
9	629.200	627.426	1.774	0.281945
10	402.700	397.230	5.470	1.358331

**Table 3:** Iao_ICP algorithm mapping error table.

Measuring Point	Measured Value (cm)	Figure Measured Values (cm)	Absolute Error (cm)	Relative Error (%)
1	284.700	283.700	1.000	0.351246
2	195.000	197.540	3.460	1.774358
3	712.200	712.363	−0.163	−0.022886
4	812.000	819.340	−7.340	−0.903940
5	271.000	270.928	0.072	0.026568
6	136.300	133.549	2.751	2.018341
7	272.300	270.116	2.184	0.802056
8	76.500	77.744	−1.244	−1.626143
9	629.200	626.829	2.371	0.376827
10	402.700	404.365	−1.665	−0.413459

## Data Availability

Data will be made available upon request from the authors.
